# A multi-centre, randomized, 3-month study to evaluate the efficacy of a smartphone app to increase caregiver’s positive mental health

**DOI:** 10.1186/s12889-019-7264-5

**Published:** 2019-07-05

**Authors:** Carme Ferré-Grau, Laia Raigal-Aran, Jael Lorca-Cabrera, Maria Ferré-Bergadá, Mar Lleixà-Fortuño, Maria Teresa Lluch-Canut, Montserrat Puig-Llobet, Núria Albacar-Riobóo

**Affiliations:** 1 Nursing Department, Campus Catalunya, Universitat Rovira i Virgili, Catalunya avenue, 35, 43002 Tarragona, Spain; 2 Nursing Department, Campus Terres de l’Ebre, Universitat Rovira i Virgili, Remolins Avenue, 13-15, 43500 Tortosa, Tarragona, Spain; 3 Computer Engineering and Mathematics Department, Campus Secelades, Universitat Rovira i Virgili, Països Catalans Avenue, 26, 43007 Tarragona, Spain; 4 Director of Territorial Health Services of Terres de l’Ebre, Sant Joan Baptista de La Salle street, 8, 43500 Tortosa, Tarragona, Spain; 5 Nursing Department, Universitat de Barcelona, de la Feixa Llarga street, s/n, 08907 L’Hospitalet de Llobregat, Barcelona, Spain

**Keywords:** Study protocol, Clinical trial, Caregiver, Mobile phone application, Positive mental health, Mhealth

## Abstract

**Background:**

To assess the effectiveness of a smartphone app-based intervention compared to a regular intervention of caregivers in primary health care institutions. The intervention is aimed at increasing positive mental health and decreasing caregiver’s burden.

**Methods/design:**

Randomized and controlled trial with an experimental group and a control group. Subjects: 108 caregivers over 18, with a minimum of 4 months of experience as caregivers. Description of the intervention: an intervention with a smartphone app (*n* = 54) or a regular intervention for caregivers (*n* = 54). Each caregiver installs a smartphone app and uses it for 28 days. This app offers them a daily activity (Monday-Friday). These activities are related to the Decalogue of Positive Mental Health, which was designed ad hoc by a group of experts. The outcomes will be the score of caregiver burden, the positive mental health and participant satisfaction. These results will be assessed after the first, third and sixth month.

**Discussion:**

The results of this study will offer evidence of the effectiveness of an intervention using a free smartphone app. If its effectiveness is proven and the results are acceptable, this could lead to a rethinking of the intervention offered to caregivers in primary care.

**Trial registration:**

Clinical Register ISRCTN14818443 (date: 24/05/2019).

## Background

Increasingly, health needs have to do with age-related chronic diseases. The World Health Organization [1] highlights the trend of the aging population with more chronic diseases which makes necessary to carry out transverse, interdepartmental and integrated actions focused on disease prevention, health promotion and person-cantered care. Moreover, all these actions must be carried out with a community and population vision. According to the Catalan Health Plan 2016–2020 [2], it is necessary to establish dynamics of collaboration between services and professionals. To this end, a context with new health needs is proposed: on the one hand, the transformation of the healthcare system and, on the other, an orientation towards a more comprehensive care that focuses on the person and the value of life expectancy in good health.

Besides the increase of aging population there is an increase of caregivers. Those are exposed to the burden associated with their caregiving activities. In fact, the Catalan Health Plan 2016–2020 has already created a project named “Self-responsibility, self-care and promotion of the autonomy of people” and one of its aims is to promote the profile of the expert caregiver. According to Bauer and Sousa-Poza [3], the most evident affectation on the part of the caregiver is in relation to their quality of psychological health. The decrease of the psychological state has a direct impact on physical health. The authors pointed out that the profile of women, spouses, and intensive caregivers represent the prototype of a caregiver profile that is most affected by caregiving tasks.

The North American Nursing Diagnosis Association (NANDA) has been providing diagnoses associated with caregivers: D00061 Fatigue of caregiver role and D00062 Risk of fatigue of caregiver role. Anarte et al. [4] used these diagnosis to evaluate the process provided by the main caregiver of the dependent patients from a primary health centre from Castellon (Spain). In this study, 32 subjects were included. The results showed that 37,5% of them suffered from fatigues and 62,5% were under the risk of suffering fatigue. In their conclusions, they highlight that the main weakness of the caregiving process is related to the problems of communication between professionals regarding the care of caregiver.

A qualitative study that also explored caregivers’ role pointed out that caregivers sometimes even behaved against to their own state of well-being. The authors emphasize the need to develop interventions to support caregivers and improve their well-being [5]. This is what takes researchers like Ferré-Grau et al. to create intervention programs based on problem solving techniques related to prevent anxiety and depression [6].

As previously stated, some authors have already presented innovative proposals that include giving support to caregivers based on new technologies. As a matter of fact, Mckechnie et al. [7] evaluated a total of 14 empirical studies of computer-mediated interventions for informal caregivers of people with dementia. As a result of this systematic review, the authors support the provision of computer-mediated intervention for caregivers of people with dementia. However, they highlight that future interventions need to be under a study protocol involving a clinical trial with a control group.

“Mastery over Dementia” is an example of this. The authors of this Internet-based intervention focused on caregivers of patients with dementia. They designed and published the protocol of [8] an Internet-based course that includes eight sessions and a booster session over a maximum period of 6 month guided by a psychologist. The results [9] of this study showed that, with a sample of 149 caregivers, the intervention was acceptable. Another example is a web-based program for informal caregivers of persons with Alzheimer by Cristancho-Lacroix et al. [10]. However, their results show that it is desirable to create a more dynamic, personalized, and social intervention instead of a web-based intervention [11].

On the basis of this evidence, this study proposes to respond to the needs set out so far. Based on a previous project in which a website for caregivers was developed (www.cuidadorascronicos.com), a protocol to develop and evaluate a smartphone app-based intervention to promote positive mental health of caregivers is presented.

### Objectives

The objective of this study is to evaluate the effectiveness of a smartphone app-based intervention compared to a standard intervention of caregivers in primary health care institutions. The intervention is aimed at increasing positive mental health and to decrease caregivers’ burden.

## Methods/design

### Study design

**Fig. 1:**
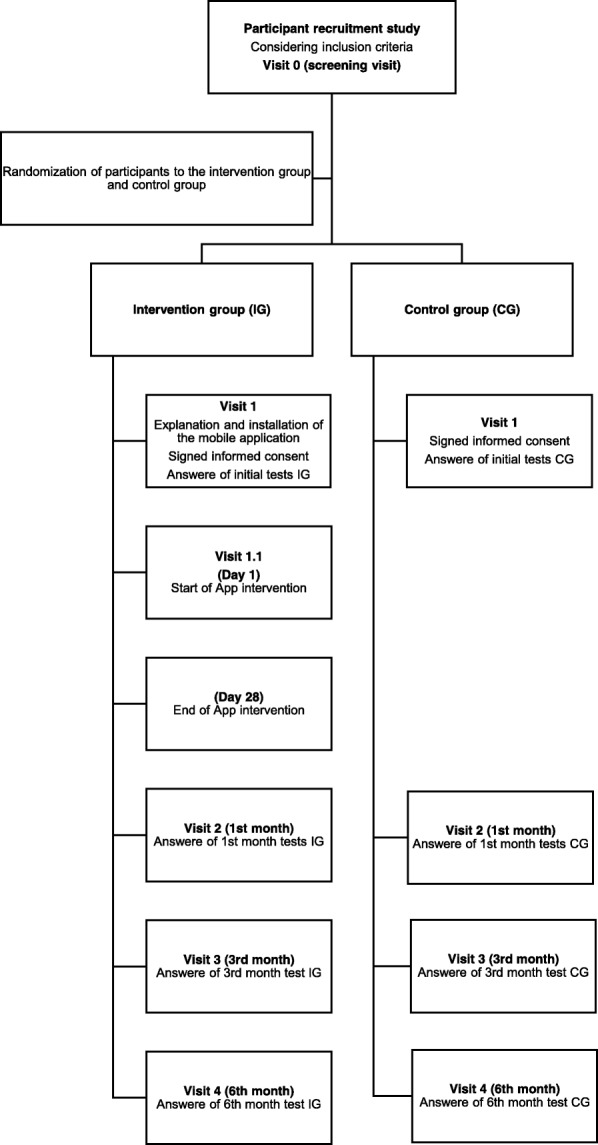
Participant Flowchart of the study

Randomized, controlled trial with an experimental group and a control group (Participant Flowchart, Fig. 1).

### Blinding

Blinding is impossible in this type of intervention. However, those responsible for.

data analysis will be blinded to group status.

### Participants and settings

Participants will be recruited from primary healthcare centres in 2 provinces of Catalonia (Tarragona and Barcelona). A randomly assignation will be done. Each primary healthcare centre that participates in this project will be provided with a copy of the study protocol.

#### Inclusion criteria

- Primary / secondary and formal / informal family caregivers of people with chronic diseases
- Over 18 years
- Minimum of 4 months of experience as caregiver
- Knowledge of Spanish or Catalan
- Use of a mobile device and WhatsApp
- Access to a mobile device with Android as operating system and with Internet access
- Signature of an informed consent

#### Exclusion criteria

- Caregivers with cognitive impairment

### Sample size

Sample size was calculated considering to an alpha risk of 0,05 and beta risk of 0,2. Considering that, to get evidential data of the results, the sample should be of 54 subjects in each group with an estimated loss of 30% during follow-up and recognizing as statistically significant a difference greater than or equal to 10.

### Screening and randomization

During the recruitment period, and until the calculated sample size has been achieved, participating care nurses who attend caregivers that meet inclusion criteria, will invite them to participate in the study (See Fig. 1). If the caregivers do not meet any exclusion criteria and agree to participate, the nurse will schedule a primary care visit (Visit 0, screening) with them.

Each participant will be assigned a code during the screening visit. The randomization unit will be the individual caregiver, assigned 1:1 to the experimental or control group. To generate the allocation sequence, the non-commercial Epidat 3.0 software will be used.

### Standard intervention for caregivers

Both study groups will receive the same standard nurse attention in primary healthcare which does not have any specific protocol. In regular attention, it is measured the caregiver burden using the scale Zarit [12] in its validation into Spanish [13].

#### Experimental group

The experimental group will receive the standard attention plus a free smartphone app. The app to be tested in the present trial has two different functions: a) an intervention in positive mental health; b) a link to an official website for caregivers. The website was developed in a previous project [14] and it is available at www.cuidadorascronicos.com.

The intervention has a duration of 28 days. During this period of time, the caregiver has the smartphone app active in his/her mobile device. Each day from Monday to Friday, the app offers him/her an activity. These activities are related to the Decalogue of Positive Mental Health by Lluch [15] which was designed ad hoc by a group of experts. After doing each activity, the caregiver can express if it has been useful or not. The app includes a motivational quote every day and it asks the caregiver “how do you feel”. During the weekend there are no activities, but the app recommends the caregiver to visit the abovementioned website. The last activity offers the caregiver the opportunity to register in this website. This allows him/her to be connected with other caregivers and to have access to any news published there.

#### Control group

Participants in the control group will only receive standard intervention for caregivers.

### Pilot test

It will be performed a pilot study with 15 participants in each group.

### Data collection

During visit 1, each participant will receive a specific dossier, which contains information about the study and their participation. There is one for the intervention group and another for the control group. The intervention group dossier includes information about the installation and information about the app. The control group dossier does not include this information. Both dossiers include information about each participation as it is explained bellow:

#### Control group

In this first visit, the initial tests of control group must be answered. These tests include two sociodemographic questionnaires: a) about the caregiver – sex, birthdate, nationality, marital status, level of studies, health problems; and b) about caretaker – type of caretaker, birthdate and sex of the patient, his/her illness, hours of care, relationship between them, helpers in caregiving and emotions related on caregiving. It also includes the scale of positive mental health by Lluch [16], and the caregiver burden [13]. Both instruments will be used in visit 2 and 3.

#### Experimental group

The experimental group has to answer questionnaires and scales as well as the control group. Besides, there is one extra questionnaire for visit 1 and visit 2. In visit 1, there is a questionnaire about the usage of technology. And in visit 2, a questionnaire about the app satisfaction.

### Follow-up

The intervention through the app does not have any direct follow-up. Nevertheless, when the caregiver gets a negative outcome from one of the smartphone app activities, the app addresses the caregiver to visit his/her nurse.

### Outcomes

The primary outcome will be a change in positive mental health and caregiver burden at the first, third, and sixth month in the experimental group, compared to the control group.

The secondary outcome will be related to the adherence and satisfaction with the app: a) adherence to the intervention, measured by frequency of using the app; b) satisfaction with activities by the responses of usefulness of each activity; c) satisfaction with smartphone app in general reported by the satisfaction questionnaire.

### Statistical analysis

The data will be analysed with SPSS version 22.0 (IBM CORP., Armonk, NY). Both groups results are going to be described by a descriptive analysis. Quantitative variables with a normal distribution will be described as means and standard deviation, or median and interquartile range otherwise, and qualitative variables as percentages and 95% confidence intervals. Possible baseline differences between trial arms will be statistically tested and further analyses will be adjusted for imbalances in the baseline scores. The primary analysis will be by intention to treat. For the primary outcomes, analyses will be corrected for multiple testing.

As sensitivity analyses, completers of all assessments will be analysed separately. Hypotheses tests will be performed at *α* = 0,05. The numeric outcomes will be analysed using analyses of variance; categorical outcomes will be analysed using *χ*^2^-tests.

## Discussion

This study will evaluate whether positive mental health improves, and burdens of care reduces on caregivers. One strength of this study is its robust design and follow-up assessment allowing the evaluation of long-term effects of the intervention. However, one of its limitations is its open design. The intervention cannot be masked; both patients and nurses in the experimental and control group will be aware that they are participating in a positive mental health intervention. This could affect to the success or failure of the intervention and could introduce bias. Randomization has been considered to help achieving a balance in sociodemographic, as well as risk factor profile. A blinded researcher will be in charge of analysing the data.

Among the strengths of the study, there is that this clinical trial has the potential to provide a validated tool to use with caregivers. A tool which could be useful to increase positive mental health. This could benefit the caregiver and even the caretaker. In addition, it has potential to decrease burdens of care. If its effectiveness is proved, this tool can be adapted to multiple frameworks.

## Data Availability

The study status is ongoing. Data will be available when the study ends (31/12/2019).
